# Aurantio‐Obtusin Suppresses Airway Inflammation and Serum ICAM‐1 Expression in Guinea Pig Allergic Asthma Model

**DOI:** 10.1002/iid3.70160

**Published:** 2025-02-28

**Authors:** Mavis Sersah Nyarko, Cynthia Amaning Danquah, Aaron Opoku Antwi, Benjamin Obukowho Emikpe, Newman Osafo

**Affiliations:** ^1^ Department of Pharmacology, Faculty of Pharmacy and Pharmaceutical Sciences Kwame Nkrumah University of Science and Technology (KNUST) Kumasi Ghana; ^2^ Department of Pathobiology, School of Veterinary Medicine Kwame Nkrumah University of Science and Technology (KNUST) Kumasi Ghana

**Keywords:** airway inflammation, Allergy, asthma, aurantio‐obtusin, intercellular adhesion molecule‐1, plant‐derived compound

## Abstract

**Introduction:**

Aurantio‐obtusin is a trihydroxyanthraquinone found in the seeds of *Cassia tora* and *Cassia obtusifolia*. Its neuroprotective, anti‐inflammatory, anti‐allergic, and antioxidant potential has been documented in multiple studies. While previous reports mention its potential as an antiasthma agent, its effects on allergen‐induced airway inflammation have not been explored.

**Method:**

Our study reports on the mechanisms by which aurantio‐obtusin exerts its effects on underlying inflammation in experimentally‐induced allergic asthma. The effect of aurantio‐obtusin pretreatment on molecular and histological changes in guinea pig lungs when challenged with aerosolized ovalbumin was assessed.

**Results:**

Our results showed that aurantio‐obtusin significantly reduced ovalbumin (OVA)‐induced increase in serum OVA‐specific immunoglobulin E (OVA‐sIgE) and intercellular adhesion molecule (ICAM)‐1. Aurantio‐obtusin further suppressed inflammatory cytokine expression (IL‐8, TNF‐α, IL‐6 and thymic stromal lymphopoietin) as well as malondialdehyde, a product of oxidative stress in bronchial lavage. The histopathological assessment showed a reduced transit of inflammatory cells and reduced deposition of collagen in the lungs of aurantio‐obtusin‐treated guinea pigs.

**Conclusion:**

Overall, the data suggests that aurantio‐obtusin mitigated ovalbumin‐induced airway inflammation by impeding the production of OVAsIgE and suppressing levels of key pro‐inflammatory cytokines. Our findings suggest that aurantio‐obtusin has potential benefits in the management of allergic airway inflammation in type 2 asthma.

## Graphical abstract

Aurantio‐obtusin mitigated ovalbumin‐induced allergic asthma by impeding the production of OVAsIgE and suppressing the expression of pro‐inflammatory cytokines. These findings support the therapeutic potential of aurantio‐obtusin for the management of asthma and other airway inflammatory disorders.

## 1 Introduction

Asthma is a heterogeneous disorder characterized by persistent inflammation of the airways, hyper‐responsiveness, and impaired airflow [1]. Specifically, Type 2 allergic asthma is a persistent inflammatory condition affecting the airways, associated with an immune response mediated mainly by T helper (Th) 2 cells and innate lymphoid cell 2 (ILC2) [2, 3, 4]. The condition involves multiple cells, airway smooth muscles, and epithelial cells working together to induce airway hyperreactivity (AHR), excessive mucus production, narrowing of the airways, and the remodeling of lung tissue [3, 5]. These processes work together to cause continuing episodes of chest tightness, shortness of breath, and wheezing in vulnerable people. Individuals experience allergy sensitization after their first contact with an allergen, which results in the formation of immunoglobulin E (IgE) antibodies. Formed IgE antibodies attach to the Fc epsilon receptor 1 (FcƐ‐RI), the IgE receptor in bronchial tissues [6, 7, 8]. On repeated exposure to the same allergen, the bound IgE antibodies cross‐link with the surface receptors, resulting in the degranulation of mast cells and release of autacoids, including vasoactive amines, prostaglandins, cysteinyl leukotrienes and an array of cytokines including interleukin IL‐4, IL‐5 and IL‐6 [9]. These mediators cause the discharge of more leukotrienes and interleukins by further activating inflammatory cells like eosinophils, basophils, lymphocytes, and alveolar macrophages, thereby sustaining the late phase of asthma. Reactive oxygen species (ROS)‐related apoptosis of epithelial cells also mediate bronchial inflammation in active asthma [10].

Asthma affects a substantial number of people worldwide, with an estimated impact on up to 300 million individuals [11]. For many patients, effective management of the disease involves a combination of inhaled corticosteroids, which help suppress inflammation, and short‐ or long‐acting β2‐adrenergic agonists, which relax constricted bronchial smooth muscles. Currently, corticosteroids are the primary drug used in controlling the underlying inflammation in asthma [12]. However, they have been documented to induce apoptosis not only in inflammatory cells like eosinophils and lymphocytes [13] but also in epithelial cells [14], leading to undesired effects. Systemic adverse effects of corticosteroids, such as morphological changes, decreased bone density, avascular necrosis, dermal thinning, adrenal suppression, immunosuppression, and heightened susceptibility to infection, among many others, have raised concerns about their long‐term use [15]. Corticosteroid resistance has also been proven to occur via reduced binding to its receptor, decline in receptor expression, and a lack of co‐repressor activity [16, 17]. Consequently, there is ongoing research aimed at identifying alternative treatments with reduced side effects and improved asthma control, including the exploration of natural sources. Notably, studies have documented the anti‐asthmatic potential of *Cassia tora* leaves in isolated goat trachea chain preparations [18]. Bioassay fractionation of the methanolic and ethanolic extracts of *Cassia tora* seeds and leaves has established aurantio‐obtusin as the major anthraquinone component. This study, therefore, assesses the effect of aurantio‐obtusin, the primary anthraquinone compound found in *Cassia tora* seeds, on airway inflammation associated with type 2 allergic asthma.

## 2 Materials and Methods

### 2.1 Materials

#### 2.1.1 Chemicals and Reagents

Aurantio‐obtusin (98%, 67979‐25‐3) (Ambeed, Illinois, USA), ovalbumin (OVA) (9006‐59‐1), and dexamethasone (50‐02‐2) were obtained from Sigma Aldrich (St. Louis, USA). Guinea pig ICAM‐1 (BL5992‐A), IL‐6 (BL6066‐A), IL‐8 (BL6033‐A), TNF‐alpha (BL3697‐A), MDA (BL6063‐A), GSH (BL6011‐A), TSLP (BL6007‐A), and OVA‐sIgE (BL4252‐A) ELISA kits (MLBio Biotechnology Company Limited, Shanghai, China).

#### 2.1.2 Animals

Guinea pigs of both sexes weighing 300–350 g were sourced from the Animal House Facility of the Faculty of Pharmacy and Pharmaceutical Sciences, KNUST and housed under standard temperature and humidity conditions (23 ± 2°C with a 12 h light‐dark cycle). Animals had unrestricted provisions for food and distilled water.

### 2.2 Methods

#### 2.2.1 Ovalbumin‐Induced Airway Inflammation (Sensitization and Challenge)

Guinea pigs were randomly selected and grouped into five (*n* = 5). Sensitization was done by intra‐peritoneal administration of 100 μl ovalbvumin solution (2 mg OVA emulsified in 10 mg aluminum hydroxide, dissolved in 10 ml normal saline) on day 0. On the 14th day, a booster dose of 100 μl ovalbumin solution (1 mg OVA in saline) was given via the intra‐peritoneal route. Sensitization was confirmed in randomly selected guinea pigs, three days prior to the aerosol challenge using the ovalbumin spin prick challenge test as described by Awortwe et al, [19]. Sensitized guinea pigs were challenged daily for 10 min with aerosolized OVA (1% OVA w/v dissolved in PBS) from day 21 to 30. Sham‐sensitization in naïve guinea pigs (*n* = 5) was done with 100 μl normal saline i.p. and challenged with PBS only. An hour before daily challenge, polyethylene glycol (PEG) (10 ml/kg, p.o), aurantio‐obtusin (10, 50, 100 mg/kg, p.o) or dexamethasone (2 mg/kg, p.o) was given to the disease control, treatment groups and positive (standard drug) control groups respectively. Aurantio‐obtusin doses were selected based on previously reported ameliorative effects on LPS‐induced pulmonary inflammation at a similar dose range [20]. Naïve guinea pigs received only normal saline 10 ml/kg, p.o. Twenty‐four (24) hours after the last exposure to ovalbumin aerosol, guinea pigs were killed by intra‐peritoneal injection with pentobarbital (80 mg/kg) and subjected to the following tests:

#### 2.2.2 Bronchoalveolar Lavage Fluid (BALF) Collection and Analysis

The tracheae of guinea pigs were carefully exposed and isolated with the lung lobes attached. The tracheal opening was clamped to avoid contamination of bronchial contents with blood. Following thorough washing of the tissue's exterior with normal saline, the clamp was removed, and bronchoalveolar fluid was collected by aspirating the cannulated trachea. The luminal contents were washed with 5 ml aliquots of PBS three times and aspirated while gently massaging the lobes [21]. The recovered fluid was centrifuged at 3000 rpm for 10 min at 4°C. The supernatant was collected and stored at −70°C. Levels of malondialdehyde (MDA), reduced glutathione (GSH), IL‐6, TSLP, IL‐8, and TNF‐α in bronchial lavage were measured using ELISA.

#### 2.2.3 Hematology and Serum Analysis

Blood was collected via the jugular vein, and full blood cell count was determined using an automated analyzer (Sysmex KX‐21N, Sysmex America Inc., Illinois, USA). Serum was separated by centrifugation (15 min, 1000 rpm). Aliquots were collected and preserved at −70°C. Serum concentration of intracellular adhesion molecule‐1 (ICAM‐1) and ovalbumin‐specific immunoglobulin E (OVA sIgE) were determined using ELISA according to the protocols outlined by the manufacturer.

#### 2.2.4 Histology

Excised lung tissues were preserved in 10% formaldehyde. After serial dehydration in varying concentrations of ethanol, clearing of the lung tissues was done with xylene in a Tissue processor (Leica Biosystems, Wetzlar, Germany), and embedded in paraffin. Transverse sections (3 μm) of the right lower lobe of the lungs were cut with a microtome (Leica Biosystems, Wetzlar, Germany) After deparaffinization and hydration in distilled water, tissue sections were stained for analysis on inflammatory cell infiltration into the airway, basement membrane thickness or collagen deposition assessment, with subsequent observation under a light microscope (Leica DM2500 M). Morphometry was done with ImageJ (version 1.50i).

##### 2.2.4.1 Airway Inflammatory Cell Infiltration

Tissue sections were stained with hematoxylin and eosin (H & E) stain and scored for cell infiltration as described by Antwi et al. [22]. A numeric score was applied as follows: 0 (no cells detected), 1 (a few cells), 2 (1 ring layer of cells), 3 (2 – 4 ring layers of cells), and 4 (ring layers of cells greater than 4) in the peribronchiolar and perivascular areas. For alveolar cell trafficking: 0 (absence of cell infiltrates and septa thickening); 1 (few infiltrates with septa thickening); 2 (profound infiltrates with septa thickening); and 3 (congested alveolar air spaces with septa thickening. A combined 11‐point score for peribronchiolar, perivascular, and alveolar cell infiltration was calculated.

##### 2.2.4.2 Assessment of Collagen Deposition

Collagen deposition in lung tissue, a measure of lung remodeling was assessed using Masson's trichrome stain. Peribronchiolar fibrosis was measured as the average area of collagen deposition (stained blue) per unit length of the basement membrane using Image J analysis tool [23]. Average‐sized bronchioles (5–7) from five random sections were assessed for each guinea pig.

#### 2.2.5 Statistical Analysis

Results are expressed as mean ± SEM. The One‐way analysis of variance (ANOVA) and Dunnet's post hoc test were used for analyses and multiple comparisons between treatment groups. All analyses were performed with GraphPad for Windows version 6 (GraphPad Prism Software, San Diego, USA).

## 3 Results

### 3.1 Bronchoalveolar Lavage Fluid (BALF) Analysis

#### 3.1.1 Effect of Aurantio‐Obtusin (AO) on Oxidative Stress

**Figure 1:**
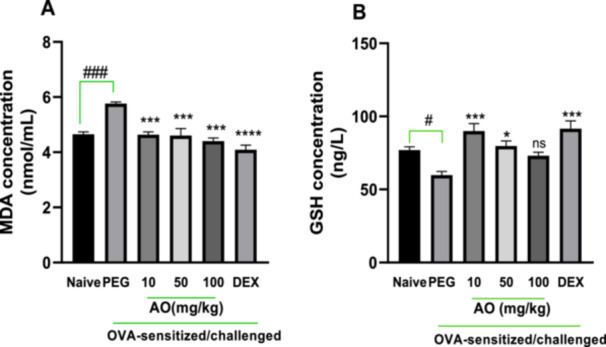
Effect of aurantio‐obtusin on oxidative stress. OVA‐sensitized guinea pigs were treated with polyethylene glycol (PEG) 10 ml/kg, dexamethasone 2 mg/kg or aurantio‐obtusin AO 10, 50, 100 mg/kg 1 h before ovalbumin aerosol challenge from day 21 to day 30. Brochoalveolar fluid was aspirated twenty‐4 h after the last challenge and analyzed with ELISA. BALF malondialdehyde (MDA) (A) and reduced glutathione (GSH) (B) levels ± SEM (*n* = 5) compared with PEG‐treated control (****p < 0.0001, ***p < 0.001, *p < 0.05) and naïve (^**#**^ *p* < 0.05 and ^###^ *p* < 0.001) with One‐way ANOVA analysis and Dunnet's test for multiple comparison.

As shown in Figure 1, the BALF analysis for polyethylene glycol (PEG)‐treated, OVA‐sensitized guinea pigs presented with an antioxidant profile suggestive of severe inflammation. The lipid peroxidation by‐product, malondialdehyde (MDA), was significantly elevated in the PEG‐treated, OVA‐sensitized, and challenged guinea pigs, measuring 5.65 ± 0.06 nmol/mL relative to the 4.65 ± 0.09 nmol/mL in the naïve group (Figure 1A). MDA levels were significantly reduced by aurantio‐obtusin at doses of 10, 50, and 100 mg/kg to 4.64 ± 0.10, 4.60 ± 0.26, and 4.40 ± 0.11 nmol/mL, respectively (Figure 1A). Also, the mean level of reduced glutathione (GSH), a primary cellular antioxidant involved in counteracting oxidative stress, was significantly reduced (59.76 ± 2.52 nmol/mL) in the PEG‐treated, asthmatic control group compared to naïve guinea pigs (76.94 ± 2.3 nmol/mL) (Figure 1B). Reduced glutathione (GSH) levels were significantly preserved in aurantio‐obtusin‐treated guinea pigs, with 10 and 50 mg/kg recording GSH levels of 89.95 ± 5.11 and 79.64 ± 3.49 nmol/mL, respectively (Figure 1B). No significant preservation was, however, observed in aurantio‐obtusin (100 mg/kg)‐treated guinea pigs. Dexamethasone treatment recorded significantly reduced MDA levels and preserved GSH, indicative of control of oxidative stress induced by the ovalbumin challenge (Figure 1A,B).

#### 3.1.2 Effect of Aurantio‐Obtusin on BALF Inflammatory Cytokines

**Figure 2:**
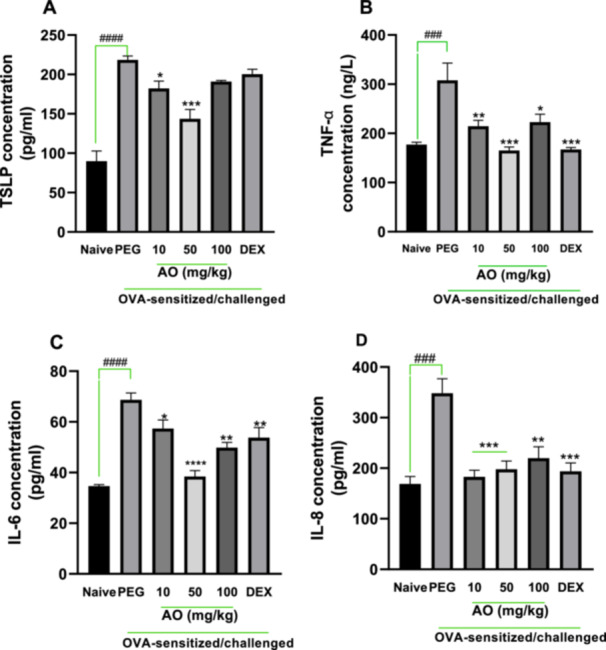
Effect of aurantio‐obtusin on BALF inflammatory cytokines. OVA‐sensitized guinea pigs were treated with polyethylene glycol (PEG) 10 ml/kg, dexamethasone or aurantio‐obtusin AO 10, 50, 100 mg/kg 1 h before ovalbumin aerosol challenge from day 21 to day 30. Brochoalveolar fluid was aspirated twenty‐4 h after the last challenge. Concentration of TSLP (A), TNF‐α (B), IL‐6 (C) and IL‐8 (D) were assessed using ELISA. Data is presented as mean ± SEM (*n* = 5) compared to PEG‐treated control (***p < 0.001, **p < 0.01, *p < 0.05) and naive (^**####**^ *p* < 0.0001 and ^**###**^ *p* < 0.001) with One‐way ANOVA analysis and Dunnet's test for multiple comparison.

As depicted in Figure 2, all cytokines examined had significantly higher levels in the BALF of the OVA‐control group. Significant reduction in levels of cytokines by 16.7%, 34.2% for TSLP (*p* < 0.5); 30.3%, 46.4%, 27.6% for TNF‐α (*p* < 0.5); 16.5%, 44.1%, 27.5% for IL‐6 (*p* < 0.5); 47.5%, 43.2%, 36.8% for IL‐8 (*p* < 0.01) was observed in aurantio‐obtusin 10, 50, and 100 mg/kg treatments, respectively. Dexamethasone decreased levels of TSLP, TNF‐α, IL‐6, and IL‐8 in BALF when compared to the asthmatic control group.

### 3.2 Blood and Serum Analysis

#### 3.2.1 Effect of Aurantio‐Obtusin on Peripheral WBC Count

**Figure 3:**
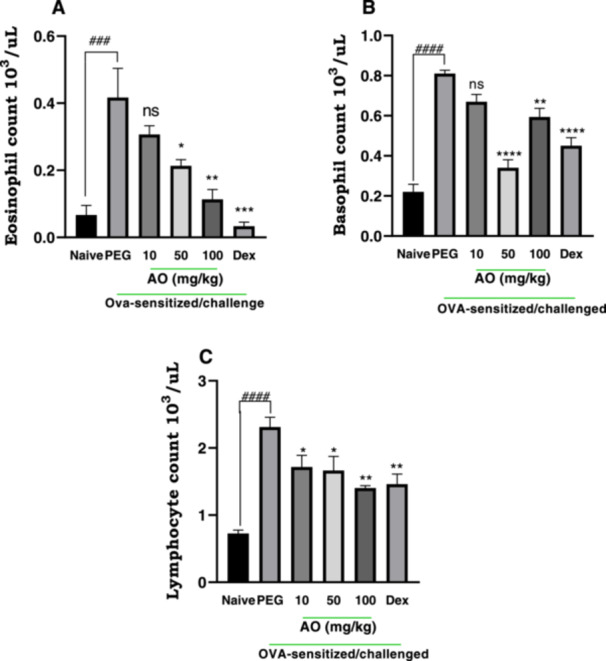
Effect of aurantio‐obtusin on peripheral WBC count. OVA‐sensitized guinea pigs were treated with polyethylene glycol (PEG) 10 ml/kg, dexamethasone 2 mg/kg or aurantio‐obtusin 10, 50, 100 mg/kg 1 h before ovalbumin aerosol challenge from day 21 to day 30. Blood was drawn for WBC count twenty‐4 h after the last challenge. Mean cell count (10^3^/µL) for eosinophils (A), basophils (B) and lymphocytes (C) ± SEM (*n* = 5) compared with PEG‐treated control (***p < 0.001, **p < 0.01, *p < 0.05) and naive (^**####**^ *p* < 0.0001 and ^###^ *p* < 0.001) using One‐way ANOVA analysis and Dunnet's test for multiple comparison.

Following aerosol exposure to ovalbumin, asthmatic control guinea pigs showed marked elevation levels in eosinophils, basophils, and lymphocytes, respectively (Figure 3). Aurantio‐obtusin (AO) treatment significantly reduced eosinophil proliferation in blood, in a dose‐dependent manner (Figure 3A). Guinea pigs treated with AO at 50 and 100 mg/kg recorded 48.80 ± 4.45% and 72.80 ± 7.11% inhibition in eosinophil counts compared to asthmatic control. The lowest dose, 10 mg/kg, however, had no significant effect on eosinophil levels (Figure 3A). AO at all three doses significantly reduced lymphocyte and basophil counts albeit in a dose‐independent manner. Aurantio‐obtusin at 10, 50, and 100 mg/kg showed 17.28 ± 4.45%, 58.02 ± 4.99%, 26.75 ± 5.35% inhibition in basophil counts (Figures 3B), and 25.55 ± 7.46%, 27.56 ± 9.08% and 38.82 ± 1.50% inhibition of lymphocyte count (Figure 3C). As expected, dexamethasone‐treated guinea pigs showed marked suppression of white blood cell counts (Figure 3), recording 92.00 ± 2.88%, 44.44 ± 4.99%, 36.36 ± 6.57% inhibition in eosinophils, lymphocytes, and basophils respectively, compared to PEG‐treated asthmatic control guinea pigs.

#### 3.2.2 Effect of Aurantio‐Obtusin on Serum Intercellular Adhesion molecule‐1 (ICAM‐1) and Ova‐SIgE Levels

**Figure 4:**
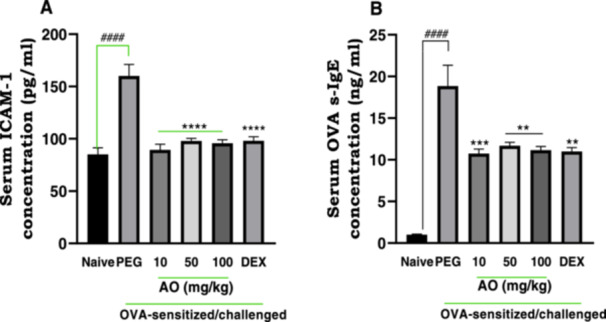
Effect of aurantio‐obtusin on serum cell adhesion molecule‐1 (ICAM‐1) levels and OVA‐sIgE levels. OVA‐sensitized guinea pigs were treated with polyethylene glycol (PEG) 10 ml/kg, dexamethasone 2 mg/kg or aurantio‐obtusin 10, 50, 100 mg/kg 1 h before ovalbumin aerosol challenge from day 21 to day 30. Serum was collected twenty‐4 h after the last challenge for determination of ICAM‐1 and OVAsIgE concentration with ELISA. ICAM‐1 (A) and OVAsIgE (B) (pg/ml) ± SEM (*n* = 5) compared with PEG‐treated control (****p < 0.0001, ***p < 0.001, **p < 0.01) and naive (^**####**^ *p* < 0.0001) using One‐way ANOVA and Dunnet's test for multiple comparison.

ICAM‐1 levels in the serum of the PEG‐treated OVA‐sensitized and challenged group were found to be significantly (*p* < 0.0001) increased (160.0 ± 11.06 pg/ml) compared to naive control group (85.08 ± 6.3 pg/ml). Aurantio‐obtusin at 10, 50, and 100 mg/kg showed significant repressive effects on the mean expression of ICAM‐1, with values of 89.46 ± 5.2 pg/ml, 97.86 ± 2.6 pg/ml, and 95.79 ± 3.3 pg/ml recorded respectively (Figure 4A).

In comparison to the naive control group (1.00 ± 0.06 ng/ml), there was a significantly higher mean expression of serum OVA‐s‐IgE (1.88 ± 0.25 ng/ml) in the PEG‐treated, ovalbumin‐challenged guinea pigs (Figure 4B). Aurantio‐obtusin at 10, 50, and 100 mg/kg significantly inhibited serum levels of Ova‐s‐IgE to 10.7 ± 0.5 ng/ml, 11.7 ± 0.4 ng/ml, and 11.1 ± 0.4 ng/ml, respectively.

The increased expression of ICAM‐1 was significantly lowered to 98.11 ± 3.80 pg/ml after treatment with dexamethasone. The average expression of OVA‐s‐IgE was also reduced significantly to 11.00 ± 0.50 ng/ml in the dexamethasone‐treated group.

### 3.3 Histology

#### 3.3.1 Effect of Aurantio‐Obtusin on Inflammatory Cell Infiltration and Basal Membrane Thickness

**Figure 5:**
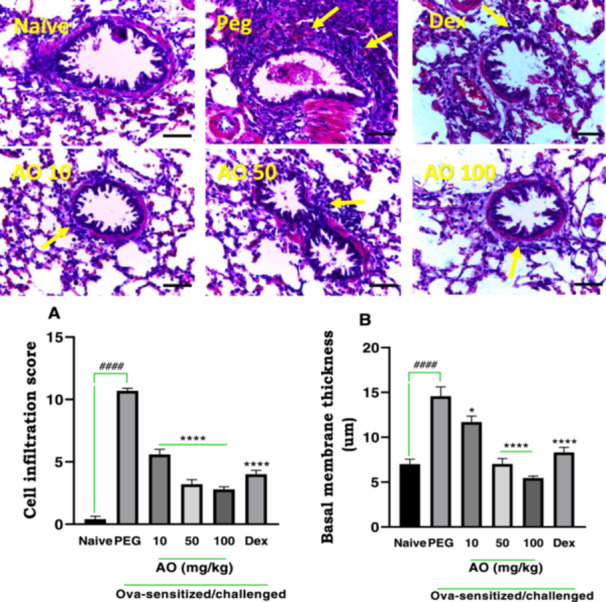
Effect of aurantio‐obtusin on inflammatory cell infiltration and basal membrane thickness. OVA‐sensitized guinea pigs were treated with polyethylene glycol (PEG) 10 ml/kg, dexamethasone 2 mg/kg or aurantio‐obtusin 10, 50, 100 mg/kg 1 h before ovalbumin aerosol challenge from day 21 to day 30. Micrographs represent H and E‐stained lung sections. Cell infiltration (A) and basement membrane thickness (B) ± SEM (*n* = 5) compared with PEG‐treated control (****p < 0.0001, *p < 0.05) and naive (^**####**^ *p* < 0.0001) using One‐way ANOVA and Dunnet's multiple comparison test. Yellow arrows indicate a ring of infiltrated peribronchiolar inflammatory cells. Scale bar indicates 100 µm.

Naïve guinea pigs (no sensitization or OVA challenge) exhibited a lung structure that appeared normal, characterized by clear alveolar spaces, minimal cellular aggregation around the bronchioles, and normal bronchial basement membrane thickness (Figure 5). Ovalbumin sensitization and challenge in the PEG‐treated group resulted in severe and extensive infiltration of inflammatory cells, forming thick peribronchiolar clusters (yellow arrows) and thickening of the bronchial basement membrane (Figure 5). Treatment with dexamethasone at 2 mg/kg reversed these pathological features. Aurantio‐obtusin at 10 to 100 mg/kg resulted in reduced inflammatory cell infiltration, less cellular congestion, and decreased thickening of the alveolar septa. Quantitative analysis revealed a score of 10.70 ± 0.20 for cell infiltration and a bronchial basement membrane width of 14.57 ± 1.06 µm in the PEG‐treated control group, which was elevated significantly when compared to the scores of 0.40 ± 0.24 and 7.00 ± 0.55 µm, for cell infiltration score and membrane thickness respectively, observed in the naïve group. Treatment with dexamethasone led to significant inhibition of cell infiltration, with a cell infiltration score of 4.00 ± 0.32, and a significant reduction in bronchial membrane thickness (8.30 ± 0.58 µm). Aurantio‐obtusin‐treated guinea pigs demonstrated a reduced bronchial basement membrane thickness compared to the PEG‐treated control group (Figure 5). The scores for cell infiltration were 5.6 ± 0.40, 3.20 ± 0.37, and 2.80 ± 0.20 at of 10, 50, and 100 mg/kg of AO, respectively. Similarly, the bronchial membrane thickness values were 11.69 ± 0.66, 7.03 ± 0.60, and 5.46 ± 0.21 µm at 10, 50, and 100 mg/kg of AO, respectively.

#### 3.3.2 Effect of Aurantio‐Obtusin on Collagen Deposition

**Figure 6:**
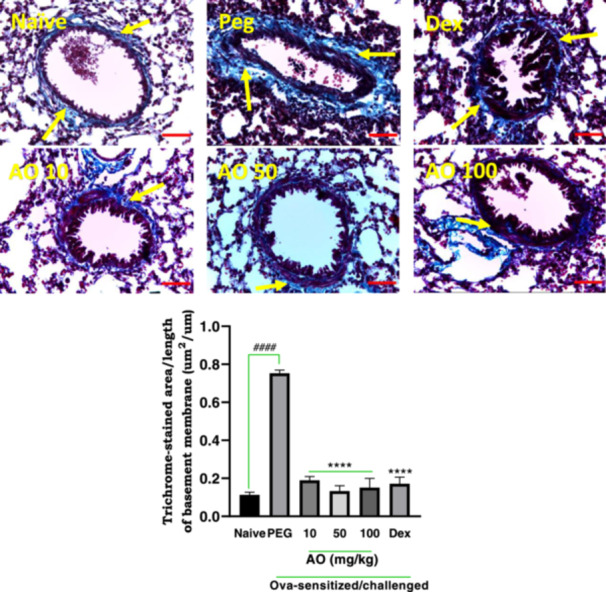
Effect of aurantio‐obtusin on collagen deposition. OVA‐sensitized guinea pigs were treated with polyethylene glycol (PEG) 10 ml/kg, dexamethasone 2 mg/kg or aurantio‐obtusin (AO) 10, 50, 100 mg/kg 1 h before ovalbumin aerosol challenge from day 21 to day 30. Micrographs represent Masson's trichrome‐stained lung sections. Collagen deposition area per basement membrane unit length ± SEM (*n* = 5) compared with PEG‐treated control (****p < 0.0001) and naïve (^**####**^ *p* < 0.0001) using One‐way ANOVA and Dunnet's multiple comparison test. Yellow arrows indicate a ring of peribronchiolar collagen deposition. Scale bar indicates 100 µm.

Sensitized guinea pigs treated with PEG exhibited significant sub‐epithelial deposition of collagen (stained blue; yellow arrows), particularly in the perivascular and peribronchiolar regions, indicating a characteristic lung remodeling feature of chronic asthma (Figure 6). In contrast, naïve guinea pigs did not exhibit any significant collagen deposition (Figure 6). The PEG‐treated group showed a collagen deposition index of 0.75 ± 0.02 µm^2^/µm, defined as the stained area per unit basement membrane length, while naïve guinea pigs showed a mean index of 0.11 ± 0.01 µm^2^/µm. Dexamethasone treatment significantly reduced the area of collagen deposition to 0.17 ± 0.04 μm^2^/μm. Aurantio‐obtusin treated animals exhibited indices of 0.19 ± 0.02 μm^2^/μm, 0.13 ± 0.03 μm^2^/μm, and 0.15 ± 0.05 μm^2^/μm, respectively, at doses of 10, 50, and 100 mg/kg indicating significant reductions compared to PEG‐treated group (Figure 6).

## 4 Discussion and Conclusion

The features of asthma include airflow obstruction, increased sensitivity of the airways, and an underlying inflammation [24]. The development of asthma is diverse, with different visible characteristics and molecular mechanisms defining various phenotypes and endotypes [25, 26]. The potential inhibitory effect of aurantio‐obtusin on disease features induced by repeated exposure to aerosolized OVA in sensitized guinea pigs was investigated in this study.

In bronchial inflammation and airway hyper‐responsiveness (AHR), some inhaled antigens bypass the clearance mechanism, penetrate the underlying epithelial layer, and evoke an immune response. This process is mimicked by the ovalbumin‐induced asthma model, exhibiting characteristics similar to allergic asthma in humans [27]. This model is thus considered to have a good predictive value in the preclinical assessment of agents with potential benefits in type 2 allergic asthma [28]. In this pathway, primed B cells, aided by the action of the cytokines IL‐4 and IL‐13, produce immunoglobulin E antibodies specific to the antigen. Hence, the measurement of this immunoglobulin E, is important in evaluating the presence and severity of asthma [29]. In this study, aurantio‐obtusin‐treatment suppressed OVA‐specific‐IgE expression in response to ovalbumin challenge, which is critical in the early stages of the allergic response and downstream, late phase, Th2‐mediated inflammatory events as well.

IgE generated in reaction to an allergen is released into the bloodstream and attaches to high‐affinity IgE receptors (FcɛRI) on mast cells and basophils. The resultant activation triggers the activation of other pro‐inflammatory cells, mainly T helper 2 (Th2) cells, and the release of pre‐stored granules containing chemicals like histamine, tryptase, chymase, eicosanoids, and free radicals [30]. In earlier reports on emodin, an anthraquinone isolated from *Rheum officinale* [31], and other promising plant‐derived compounds such as genipin [32] and norisoboldine [33], inhibition of serum ovalbumin‐specific antibodies was central to the attenuation allergen‐induced early and late phase ‘asthma‐like’ manifestations. This was consistent with our findings.

The movement of inflammatory cells into the lungs is associated with the onset of inflammation in airway hyperresponsiveness [34]. Elevations in the eosinophil count in blood, and bronchial lavage are indicative of disease severity in allergic asthma. This is particularly prominent in the eosinophilic asthma subtype [35, 36]. They release a number of important proteins and different mediators that help amplify allergic reactions and the airway remodeling process [37]. By encouraging eosinophilic inflammation and mucus production, basophils contribute significantly to maintaining the late phase of the allergic response [38, 39]. Ovalbumin sensitization and challenge in this study induced elevated inflammatory cells in the blood of the PEG‐treated animals. Aurantio‐obtusin effectively mitigated this rise, just as occurred in dexamethasone treatment. Several cytokines contribute to the pathophysiology of chronic allergic asthma. These elements all play a role in the emergence, development, and maintenance of persistent airway inflammation [40, 41, 42]. In this study, aurantio‐obtusin significantly inhibited the expression of IL‐6, reduced serum Ova‐specific IgE and accordingly showed very minimal eosinophil proliferation. IL‐6 in the airways has been associated with a decline in central airway function [43]. When combined with TGF, it improves the development of Th17 cells and inhibits the differentiation of Th1 cells while encouraging the generation of IL‐4 during the differentiation of Th2 cells [44]. Also, IL‐8, a CXC chemokine strongly expressed in inflammatory conditions has its gene regulated by nuclear factor κB (NF‐κB), a key target for the suppression of IL‐8 production mediated by corticosteroids. The significant reduction in the expression of IL‐8 by aurantio‐obtusin in this study reiterates the possible modulation of the nuclear factor κB pathway as reported by Hou et al. [45]. The effect of aurantio‐obtusin on these cytokines and chemokine linked to airway inflammation gives compelling evidence that aurantio‐obtusin holds prospects in the management of allergic airway defects.

The innate immunity within the lungs includes the bronchial epithelial cells, which act against inhaled allergens [7]. Epithelial cells release a variety of mediators after being stimulated by antigens or pro‐inflammatory cytokines, one of which is thymic stromal lymphopoietin (TSLP), which is important in initiating allergic airway inflammation. Many cells mediating inflammation in asthma are stimulated by TSLP [46]. In this study, aurantio‐obtusin significantly inhibited the expression of TSLP, demonstrating its potential as a suppressor of TSLP‐mediated inflammation. Our observations support the findings by Lee et al. [47] and Li et al. [48]. They demonstrated that neutralization of TSLP or a reduction in its expression was linked with reduced airway inflammation and reduced disease severity in experimentally‐induced allergic asthma.

Elevation in the levels of intercellular adhesion molecule‐1 (ICAM‐1) has been linked to eosinophil adherence to bronchial epithelial and vascular endothelial cells, which results in bronchial inflammation, according to a number of studies [49, 50]. Individuals with asthma exhibit elevated expression of ICAM‐1 [51]. Notably, this study found that aurantio‐obtusin treatment significantly decreased ICAM‐1 levels at all tested doses. These results imply that aurantio‐obtusin may decrease the heightened inflammation brought on by elevated ICAM‐1 expression. As seen in our study, other treatments that have been reported to suppress ICAM‐1 expression eventually mitigated cell‐mediated lung tissue damage and resultant remodeling [52, 53]. Oxidative stress significantly contributes to cell membrane disruption, protein and DNA damage in asthma [54]. Inflammation, remodeling, and the severity of asthma are thought to be influenced by a distortion in the balance between reactive oxygen species and antioxidant defense mechanisms [55, 56]. There is evidence from numerous research that antioxidant consumption and lung function are positively correlated. Measurements of oxidative stress markers in breath condensates of both animal and human investigations [57] and bronchial fluids [58] relate positively to disease progression. The results of this study's investigation of guinea pig bronchoalveolar lavage fluid (BALF) showed that treatment with aurantio‐obtusin mitigated the levels of oxidative stress.

Guinea pigs given aurantio‐obtusin recorded preserved levels of reduced glutathione comparable to naïve control guinea pigs. In sustained inflammatory states, prolonged reactive oxygen species activity depletes antioxidant defenses such as reduced glutathione [59]. Preserved levels in aurantio‐obtusin‐treated guinea pigs suggest significant suppression of these processes. Consistent with this, MDA, a byproduct of lipid peroxidation was reduced in aurantio‐obtusin‐treated guinea pigs. This is key to understanding the effect of aurantio‐obtusin, since sufficient glutathione levels are linked with effective free radical scavenging, regulation of DNA synthesis and repair, as well as prevention of early phase hypersensitivity in asthma. Several studies also point to improved symptoms in both clinical and experimental asthma when glutathione levels are adequately preserved [60, 61, 62, 63].

We observed some nonlinear responses at the highest dose of auratio‐obtusin 100 mg/kg for cytokine, cell proliferation, and oxidative stress marker measurements. Similar trends were observed in our earlier report on the anti‐rhinitis effects of aurantio‐obtusin [64]. Hormetic effects of this nature have been reported for plant‐derived compounds and extracts in vivo and in vitro [65, 66, 67, 68, 69]. These have been attributed to the presence of drug target subtypes with opposing effects and adaptive molecular responses in biological systems upon exposure to higher doses of xenobiotics [65, 70]. The specific events in our case, however, require further investigation.

In cases of persistent and uncontrolled airway inflammation, characteristic histological features observed include mucous plugging, epithelial desquamation and hyperplasia, collagen production, and sub‐epithelial fibrosis, as well as smooth muscle hypertrophy and hyperplasia [6, 71]. In this study, airway remodeling, specifically collagen deposition was evaluated using Masson's trichrome stain. In areas around the bronchioles and blood vessels, asthmatic control guinea pigs showed significant collagen‐positive staining, as expected. These results indicate the evidence of airway remodeling. However, aurantio‐obtusin at all doses significantly attenuated these manifestations of airway remodeling. Overall, these effects, considered alongside findings by She et al. [72], which demonstrated aurantio‐obtusin involvement in calcium pathways associated with bronchorelaxation in mice, suggest a significant inhibitory effect on both acute or early‐phase and late‐phase pathophysiology of asthma.

In conclusion, the results clearly indicate that aurantio‐obtusin exerts a potent suppressive effect on various key aspects of type 2 asthma pathogenesis such as airway inflammation, Th2 cytokine release, OVA‐specific IgE levels, and eosinophil recruitment. These findings indicate that aurantio‐obtusin has the potential to be a therapeutic alternative for the management of airway inflammation in Type 2 asthma.

## Author Contributions

**Mavis Sersah Nyarko:** conceptualization, data curation, methodology, writing–original draft, writing–review and editing. **Cynthia Amaning Danquah:** formal analysis, supervision, writing–review and editing. **Aaron Opoku Antwi:** conceptualization, methodology, writing–original draft. **Benjamin Obukowho Emikpe:** methodology, supervision, writing–review and editing. **Newman Osafo:** investigation, supervision, writing–original draft, writing–review and editing.

## Ethics Statement

The KNUST Ethics Committee (Approval No. KNUST 0056) reviewed and approved all protocols used in this study. Animal Welfare Regulations (USDA 1985; US Code, 42 USC § 289 d) and the Public Health Service Policy on Humane Care and Use of Laboratory Animals (PHS 2002) were followed in animal handling procedures. There are no studies on human participants in this article.

## Conflicts of Interest

The authors declare no conflicts of interest.

## Data Availability

The datasets generated and analyzed during the current study are available from the corresponding author upon reasonable request.
